# Generation and characterization of human U-2 OS cell lines with the CRISPR/Cas9-edited protoporphyrinogen oxidase IX gene

**DOI:** 10.1038/s41598-022-21147-x

**Published:** 2022-10-12

**Authors:** Zora Novakova, Mirko Milosevic, Zsofia Kutil, Marketa Ondrakova, Barbora Havlinova, Petr Kasparek, Cristian Sandoval-Acuña, Zuzana Korandova, Jaroslav Truksa, Marek Vrbacky, Jakub Rohlena, Cyril Barinka

**Affiliations:** 1 Laboratory of Structural Biology, Institute of Biotechnology of the Czech Academy of Sciences, BIOCEV, Prumyslova 595, Vestec, 25250 Czech Republic; 2 Laboratory of Cellular Metabolism, Institute of Biotechnology of the Czech Academy of Sciences, BIOCEV, Prumyslova 595, Vestec, 25250 Czech Republic; 3 Faculty of Science, Charles University, Vinicna 5, Prague, 12108 Czech Republic; 4 Czech Centre for Phenogenomics, Institute of Molecular Genetics of the Czech Academy of Sciences, BIOCEV, Prumyslova 595, Vestec, 25250 Czech Republic; 5 Laboratory of Tumour Resistance, Institute of Biotechnology of the Czech Academy of Sciences, BIOCEV, Prumyslova 595, Vestec, 25250 Czech Republic; 6 Laboratory of Bioenergetics, Institute of Physiology of the Czech Academy of Sciences, Videnska 1083, Prague, 14220 Czech Republic; 7 First Faculty of Medicine, Charles University, Katerinska 32, Prague, 12108 Czech Republic

**Keywords:** Oxidoreductases, Proteomics, Mechanisms of disease, Experimental models of disease, Cell culture

## Abstract

In humans, disruptions in the heme biosynthetic pathway are associated with various types of porphyrias, including variegate porphyria that results from the decreased activity of protoporphyrinogen oxidase IX (PPO; E.C.1.3.3.4), the enzyme catalyzing the penultimate step of the heme biosynthesis. Here we report the generation and characterization of human cell lines, in which PPO was inactivated using the CRISPR/Cas9 system. The PPO knock-out (PPO-KO) cell lines are viable with the normal proliferation rate and show massive accumulation of protoporphyrinogen IX, the PPO substrate. Observed low heme levels trigger a decrease in the amount of functional heme containing respiratory complexes III and IV and overall reduced oxygen consumption rates. Untargeted proteomics further revealed dysregulation of 22 cellular proteins, including strong upregulation of 5-aminolevulinic acid synthase, the major regulatory protein of the heme biosynthesis, as well as additional ten targets with unknown association to heme metabolism. Importantly, knock-in of PPO into PPO-KO cells rescued their wild-type phenotype, confirming the specificity of our model. Overall, our model system exploiting a non-erythroid human U-2 OS cell line reveals physiological consequences of the PPO ablation at the cellular level and can serve as a tool to study various aspects of dysregulated heme metabolism associated with variegate porphyria.

## Introduction

Heme is a prosthetic group and a signaling molecule that is indispensable for the life of all aerobic organisms. It serves as a metallocofactor for a host of proteins including hemoglobin, myoglobin, catalases, peroxidases, and cytochromes. Therefore, heme plays a crucial role in essential metabolic processes such as enzyme catalysis, cell respiration, photosynthesis, oxidative stress response, iron homeostasis, cell cycle progression and apoptosis (reviewed in ref.^1–3^). Heme is synthesized in a multi-stage process that involves 8 enzymatic reactions and glycine, succinyl coenzyme A, molecular oxygen, and iron as substrates^4^. The first and last three steps of heme biosynthesis are localized to mitochondria, while the remaining reactions are performed in the cytoplasm^3,5^.

Heme synthesis, trafficking, utilization, and degradation must be precisely controlled and tightly regulated since high concentration of the heme and/or its intermediates is associated with cellular toxicity. The main regulatory mechanism acts at the first step of biosynthesis where 5-aminolevulinic acid synthase (ALAS) is negatively regulated by heme at multiple levels (reviewed by^6^). For degradation, heme is catabolized to biliverdin, carbon monoxide and free iron by heme oxygenases, mostly localized in the endoplasmic reticulum membrane^7,8^. Transport of heme from mitochondria to other cellular compartments as well as shuttling of its intermediates between mitochondria and the cytosol is still poorly understood. Due to its low solubility and its propensity to generate cytotoxic reactive oxygen species, cellular heme is stored and transported by a variety of chaperons, transporters and carrier proteins^9,10^. Cells can also uptake extracellular heme by either heme transporters^11–13^ or through the endocytosis of extracellular hemoproteins^14–16^.

Defects in the activity of individual enzymes of the heme biosynthetic pathway usually lead to accumulation of distinct heme precursors and these deficiencies are then clinically manifested as rare inherited disorders collectively known as porphyrias^17^. Phenotypically, porphyrias can be divided into acute or cutaneous porphyrias that primarily affect either the nervous system or the skin, respectively (reviewed in^2,18–20^). Variegate porphyria (VP), an autosomal dominant disease with incomplete penetrance, is characterized by photosensitivity and acute neurovisceral attacks and it results from the deficiency of protoporphyrinogen oxidase IX (PPO; E.C.1.3.3.4), the enzyme catalyzing the penultimate step of the porphyrin-heme biosynthetic pathway^21–25^. Over 180 mutations in the PPO sequence have been described in the literature that are linked to the VP^20,26–30^. The *PPOX* gene is localized to the chromosome 1q23^31,32^ and contains 13 exons^31,33^. Mature PPO of 477 amino acids comprises flavin adenine dinucleotide as its essential co-factor and catalyzes oxidation of protoporphyrinogen IX to protoporphyrin IX, which serves as a common intermediate for synthesis of heme as well as chlorophyll^34–37^. In the mitochondrial matrix, PPO is believed to form a heterotetrameric complex with ferrochelatase, the ultimate enzyme of the heme biosynthetic pathway^38,39^.

PPO enzymatic activity is essential for animals as well as plants—disruption of the *PPOX* gene results in embryonal lethality in animals^40^, and plant PPOs are targeted by herbicides to control weeds to sustain and improve agricultural production^41,42^. While enzymatic and structural characteristics of purified human PPO are well described in vitro, the understanding of consequences of its absence at the cellular level are virtually missing. To evaluate effects of PPO ablation on cell physiology, we used the **c**lustered regularly interspaced short palindromic repeats (CRISPR)/Cas9 system to establish PPO knock-out U-2 OS cell lines and characterized them in detail.

## Results

### PPO-deficient cell lines are viable

**Figure 1:**
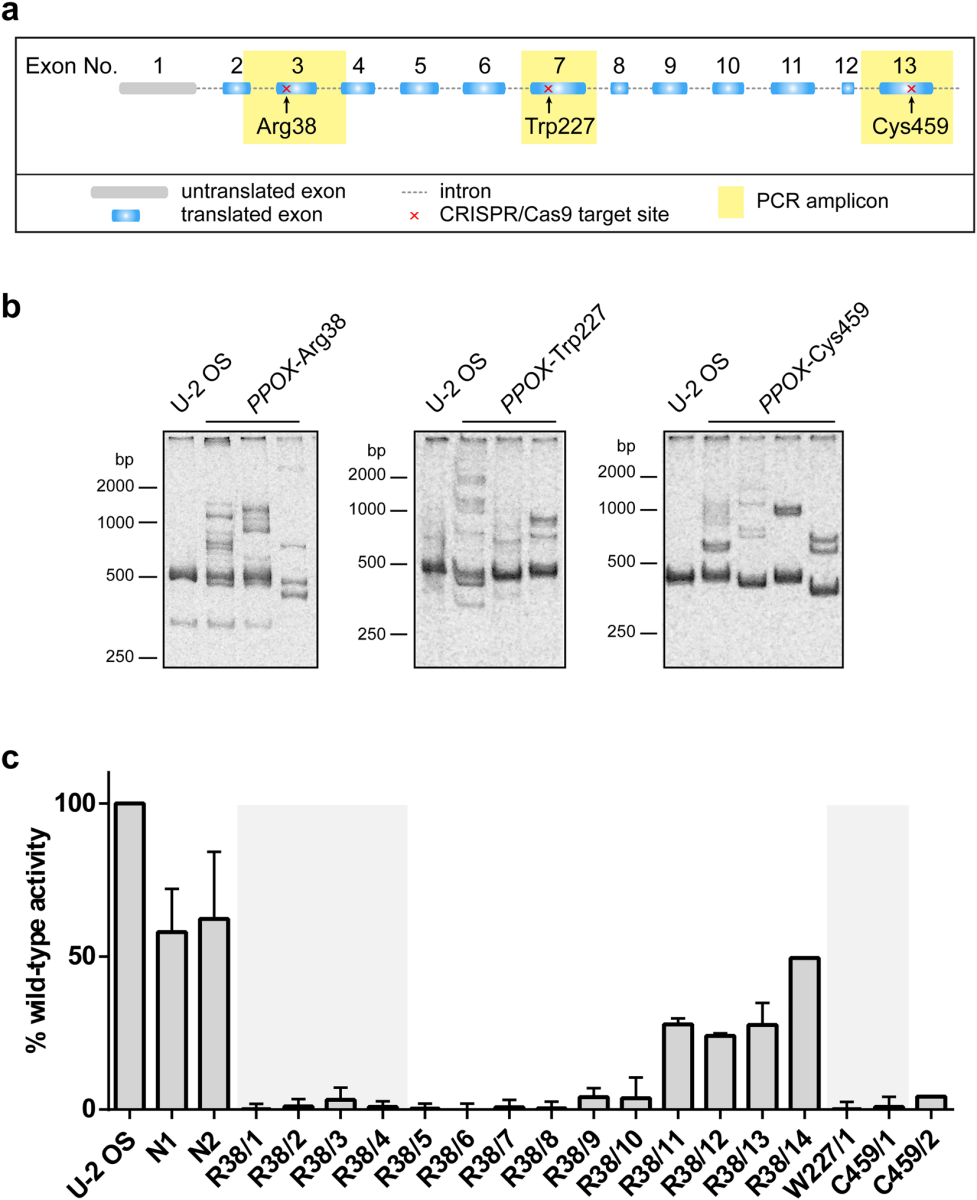
Establishing PPO-KO cell lines by CRISPR/Cas9 editing of the *PPOX* gene. *(***a**) Schematic representation of *PPOX* gene disruption. Positions of CRISPR/Cas9 target sites are marked by red crosses. Sequences amplified by PCR for detection of heteroduplexes and allele genotyping are highlighted in yellow. (**b**) Analysis of *PPOX* allele heteroduplexes from CRISPR/Cas9 edited cell lines. Regions encompassing CRISPR recognition sites were PCR-amplified, annealed, and separated by PAGE with GelRed staining. Representative samples of CRISPR/Cas9 clones of *PPOX*-Arg38, *PPOX*-Trp227, and *PPOX*-Cys459 loci are shown. (**c**) The PPO enzymatic activity in cell lysates of PPO-KO and control clones was determined by quantifying the conversion of protoporphyrinogen IX to protoporphyrin IX. PPO oxidase activity in individual clones was normalized to the total protein content and is shown as a fraction of the activity of the U-2 OS parental cell line. Cell clones selected for further analysis are highlighted by grey background. Data represent mean (± S.D.); n = 3.

CRISPRs targeting three distinct loci of the human *PPOX* gene (Fig. 1a)—Arg38 (exon 3), Trp227 (exon 7), and Cys459 (exon 13)—were designed using CRISPOR Design Tool software^43^. Guide RNAs (Table S1) were cloned into the px458 plasmid harboring the GFP-fluorescence marker, and the resulting plasmids transfected into U-2 OS cells using the GeneX*Plus* transfection reagent. Approximately 200 CRISPR/Cas9-transfected GFP-positive clones were established by the combination of fluorescence-activated single cell sorting and limited dilution resulting in single cell colonies.

Following colony expansion, the primary screen for evaluation of the target sequence modification by CRISPR/Cas9 was carried out by PCR-based genotyping. To this end, the targeted loci were amplified using matching sets of primers (Table S2) and annealed amplicons separated by polyacrylamide gel electrophoresis. While PCR amplicons from the control wild-type parent U-2 OS cells migrate as a single band, the migration pattern of heteroduplexes, which would be formed by annealing PCR products of different genomic DNA templates modified by the non-homologous recombination repair events upon CRISPR/Cas9 cleavage, is manifested by the presence of several bands with mobilities different from the wild-type allele (examples of heteroduplexes shown in Fig. 1b and S1). Out of 61 heteroduplex-positive cell clones, we selected 14, 1, and 2 CRISPR/Cas9 clones (PPO-KO) for *PPOX*-Arg38, *PPOX*-Trp227, and *PPOX*-Cys459 loci, respectively, for further characterization. The clones were selected based on the prominent presence of heteroduplexes and simultaneous depletion of the signal of the band corresponding to the original wild type allele.

### PPO enzymatic activity is absent from clones with ablated PPO

The presence of PCR heteroduplexes points towards modifications to the *PPOX* encoding DNA sequences, but it did not allow us to determine whether these mutations were manifested in the changes of the PPO function. We thus first determined PPO enzymatic activity in cell lysates from individual U-2 OS clones (Fig. 1c). For parent wild-type cells, the PPO activity was 0.22 pmol/mg of total protein/sec. Out of 17 clones tested, almost absent PPO activity (< 5% of the wild-type activity) was observed in 13 clones, while > 20% of the wild-type activity was detected in four clones. The residual PPO enzymatic activity suggests that the latter cultures either do not originate from a single cell but contain a mixture of CRISPR-edited and parent cells, or these might be cells, where only a single allele is mutated. Six clones lacking the PPO enzymatic activity were selected for further experiments (highlighted by the gray background; Fig. 1c) including four *PPOX*-Arg38 clones (R38/1–R38/4), and a single clone for each of the *PPOX*-Trp227 (W227/1) and *PPOX*-Cys459 (C459/1) locus.

In addition to the parental U-2 OS cell line, we further included two other controls to increase rigor of our experimental findings and to account for possible effects of clonal variability. The N1 control culture was prepared by CRISPR/Cas9-driven editing (GFP knock-in) of the gene encoding histone deacetylase 6, which is not known to be associated with the heme metabolism in any aspect. The N2 control is a PPO CRISPR/Cas9 clone where no heteroduplexes were detected by PCR analysis and the presence of intact *PPOX* alleles was confirmed by genotyping (see below).

### Genotyping of CRISPR/Cas9 knock-out clones reveals the presence of two PPOX-encoding alleles

To investigate the exact nature of mutations in the six PPO-KO and the N2 control clone, we isolated the genomic DNA, amplified targeted loci by PCR, subcloned them into a pUC19 vector and analyzed by Sanger sequencing. In all clones, except for R38/1, exactly two copies/alleles of the *PPOX* gene were identified by sequencing. Our data thus corresponds well with previously published finding that U-2 OS cells contain 2 to 3 copies of chromosome 1, the carrier of the *PPOX* gene^44,45^. While the N2 clone had both *PPOX* encoding alleles intact, insertions, deletions, and/or mutations, resulting in disruptions to the *PPOX* sequence and reading frame, were identified in both alleles of remaining six clones (Table 1). For the R38/1 clone, only one CRISPR/Cas9-edited allele was found suggesting that the second allele contained a large CRISPR/Cas9-induced deletion that prevented amplification of the target site by PCR.

**Table 1:** Detailed description of nucleotide sequence modifications identified in individual *PPOX* alleles edited by CRISPR/Cas9.

Clone	Allele 1	Allele 2
N2	Intact	Intact
R38/1	78 nt deleted	Not identified
R38/2	12 nt inserted, 3 nt mutated	100 nt mutated
R38/3	10 nt deleted—early STOP codon	36 nt deleted
R38/4	19 nt deleted—early STOP codon	17 nt deleted—early STOP codon
W227/1	2 nt inserted—early STOP codon	7 nt deleted—early STOP codon
C459/1	57 nt deleted	69 nt deleted

*nt *nucleotide.

### Quantities of cellular heme and heme precursors are reversed in PPO-KO cells

**Figure 2:**
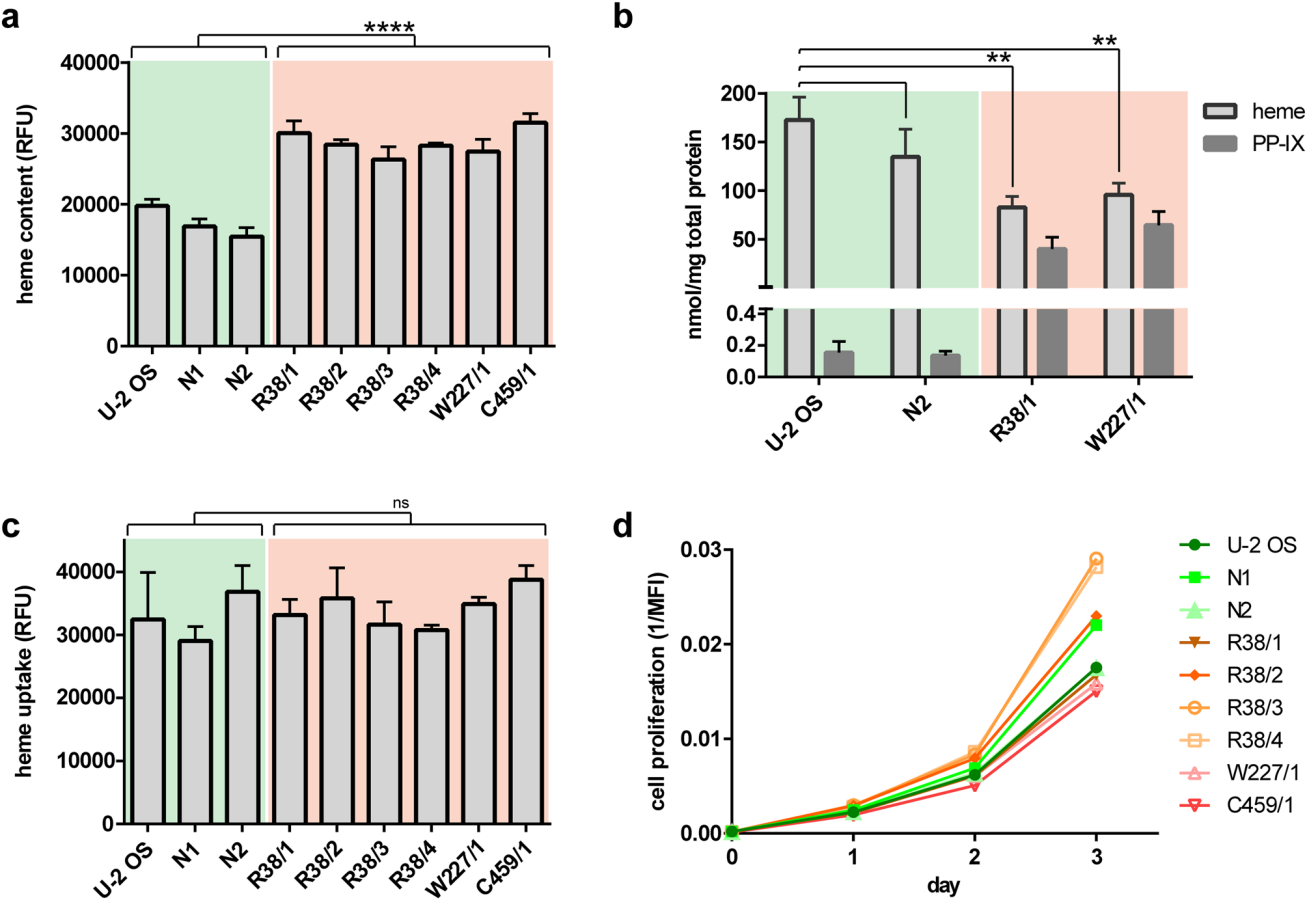
Characterization of PPO-KO cell lines. (**a**) Content of intracellular heme determined by iron-chelation assay. Statistical significance was calculated by unpaired parametric t-test with Welch’s correction and assigned by **** (P < 0.0001). Data represent mean (± S.D.); n = 3. Control samples and PPO-KO clones are highlighted by green and orange background, respectively. (**b**) Content of intracellular protoporphyrinogen/protoporphyrin IX and heme determined by RP-HPLC. Statistical significance was calculated by one-way ANOVA and assigned by ** (P < 0.01). Data represent mean (± S.D.); n = 3. (**c**) Uptake of Zn-protoporphyrin (Zn-PP; fluorescent heme analog) by studied cell lines. Fluorescence of intracellular Zn-PP was determined following 3-h incubation of cells with 60 µM Zn-PP. Statistical significance was calculated by unpaired parametric t-test with Welch’s correction and assigned by ns (non-significant; P > 0.05). Data represent mean (± S.D.); n = 2. (**d**) Proliferation rate of studied cells was determined by quantifying intracellular concentration of the fluorescent CFSE reagent by flow cytometry. The median of fluorescence intensity (MFI) is plotted against cultivation time. Data represent mean (± S.D.); n = 2.

Since we disrupted PPO that represents indispensable part of heme biosynthesis, we expected that heme concentration would be lower in PPO-KO clones. We thus first determined the heme content in 6 + 3 (KO and control) cell lines using a standard iron-chelation assay^46^. In this assay, nonfluorescent heme is converted to fluorescent porphyrin by iron chelation using oxalic acid, thus the fluorescence signal should correspond to the amount of intracellular heme. To our surprise and in contrast to expected results, the heme content was reproducibly 1.5-fold higher in PPO-KO cells compared to control cell lines (Fig. 2a).

To verify the above unforeseen results and to obtain more quantitative data on the cellular content of both heme and heme precursors, we used reversed-phase high-performance liquid chromatography (RP-HPLC) to quantify the content of heme and protoporphyrinogen/protoporphyrin IX (PP-IX) in cell extracts from two PPO-KO clones and two control cell lines (Fig. 2b, S2 and S3). It shall be noted, that during sample preparation protoporhyrinogen IX is spontaneously oxidized to protoporphyrin IX. The RP-HPLC assay is thus unable to differentiate cellular protoporphyrinogen IX from protoporphyrin IX and only the combined amount of both precursors is measured. Clones R38/1 and W227/1 contained 83 ± 11 and 96 ± 12 nmol heme/mg total protein, respectively, corresponding to approximately twofold decrease in comparison to control cells that contained 173 ± 23 nmol heme/mg protein. At the same time, > 200-fold increase in the PP-IX levels was observed in R38/1 (40 ± 12 nmol/mg protein) and W227/1 (65 ± 14 nmol/mg protein) clones compared to the parent U-2 OS cells (0.15 ± 0.07 nmol/mg protein). Overall, our data show that the absence of PPO enzymatic activity leads to the massive accumulation of PP-IX (more likely the protoporphyrinogen IX substrate) and corresponding decrease in the total heme content, leading to pronounced physiological defects (see below). Additionally, the comparison of two experimental approaches for heme quantification suggests that the standard iron-chelation assay might not be the best suited for instances where the heme synthesis/degradation pathways are compromised, and more sophisticated approaches are required to obtain reliable data.

To confirm that increased levels of PP-IX found in PPO-KO cells are not an artifact of cell cloning, we treated cells by hemin for 20 h and then we quantified levels of heme and PP-IX by RP-HPLC. Our data shows that treated cells accumulated heme in a concentration–dependent manner (Fig. S4a), with the concomitant decrease in the PP-IX levels (up to 0.1 fold of the original value depending on hemin concentration; Fig. S4b).

### Cellular heme uptake is identical in both KO and control cell lines

When the heme synthetic pathway is compromised, cells can offset the lower heme synthesis rate by its import from the culture medium. We thus used cell lines reported here to examine how the heme-synthesis deficiency influences the heme uptake. The heme import was quantified using Zn-protoporphyrinogen IX (Zn-PP), a fluorescent heme analog^47^. To this end, cells were cultured in the presence of Zn-PP for three hours and the intracellular Zn-PP fluorescence intensity quantified. No significant difference in the Zn-PP cellular content was found between the control and PPO-KO cell lines suggesting that lower intracellular concentrations of heme did not lead to more avid heme uptake from the cultivation media (Fig. 2c).

### Cell proliferation is not influenced by PPO ablation

To evaluate the impact of PPO deletion on cell proliferation, the growth rate of PPO-KO and control cell lines in RPMI1640 medium supplemented with 10% FBS was assessed using the carboxyfluorescein succinimidyl ester (CFSE) labeling protocol, where the cell doubling rate negatively correlates with the intensity of intracellular fluorescence of the fluorophore^48^. Surprisingly, the growth rate of PPO-KO clones was similar to that of control cell lines suggesting that the heme synthesis pathway is not required for the cell growth under “traditional” cultivation conditions in vitro, where all nutrients are available in excess (Fig. 2d). Cellular heme content and uptake as well as cell proliferation is likely affected by the heme present in the serum-supplemented cultivation media. Consequently, the use of serum-free media could bring additional insights into physiological aspects studied here. Unfortunately, though, all attempts to cultivate U-2 OS cells and derived clones in a serum-free medium were unsuccessful.

### Proteome analysis reveals increased levels of ALAS-1 as well as decreased levels of heme-containing proteins

**Figure 3:**
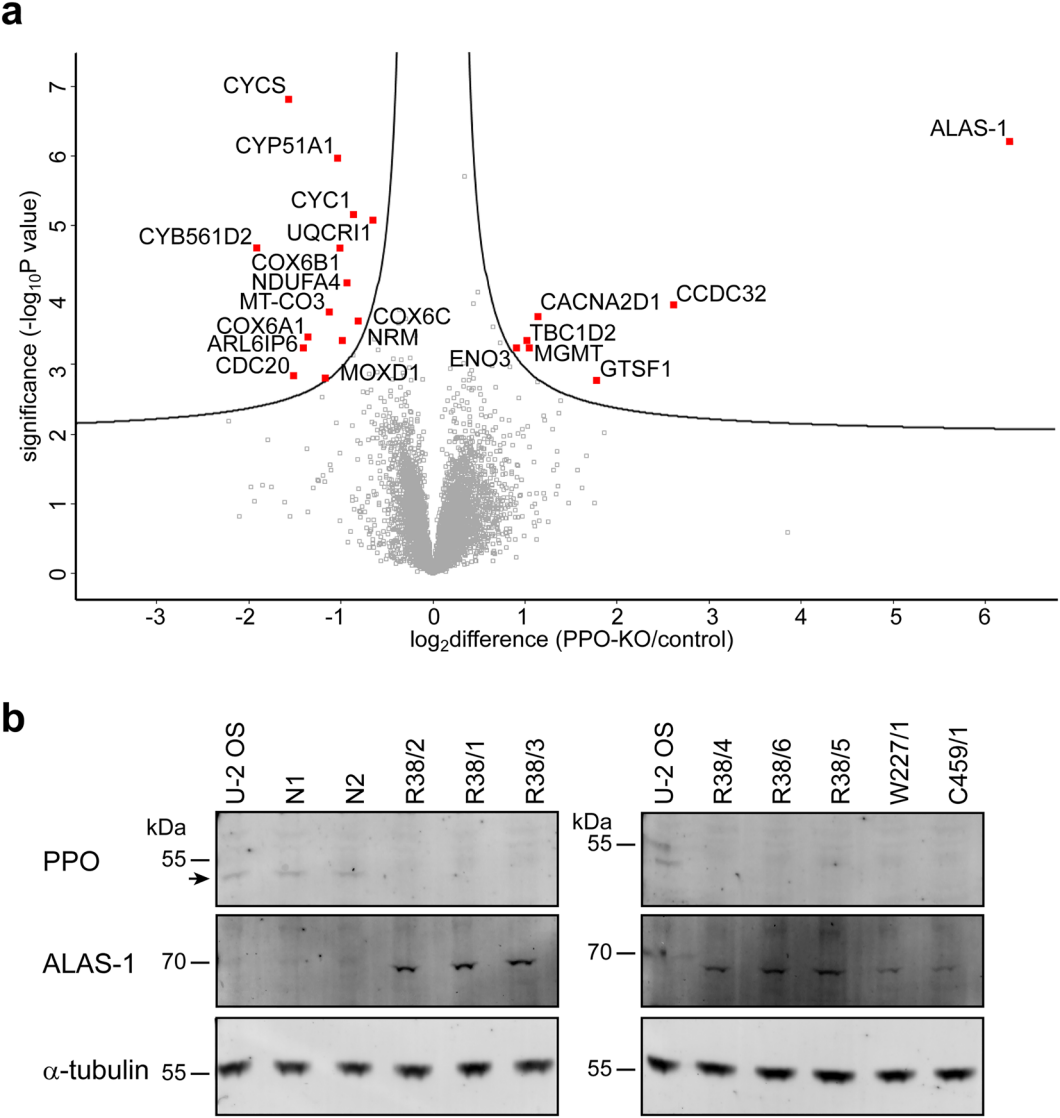
Analysis of protein levels in PPO-KO cells. (**a**) Volcano plot of significantly differentially abundant proteins in PPO-KO cells compared to control cell lines. Cell lysates of two control (U-2 OS and N2) and two PPO-KO (R38/1 and W227/1) clones were digested with Lys-C/trypsin and resulting peptides identified and quantified using mass spectrometry-label free quantification analysis. In the volcano plot, the − log_10_ (Benjamini–Hochberg corrected P value) is plotted against the log_2_ (fold change: PPO-KO/control). Proteins significantly dysregulated in PPO-KO clones (localized above the curves drawn in Perseus with parameters FDR 0.05, s0 0.1.) are marked in red. (**b**) Expression levels of PPO (position marked by arrow) and ALAS-1 were determined by Western blotting of cell lysates separated by SDS-PAGE. Alpha-tubulin was used as a loading control.

Since the ablation of PPO perturbed the heme synthesis pathway, we asked how these changes impact steady-state levels of the cellular proteome. To this end, we used mass spectrometry-label free quantification analysis (MS-LFQ) to obtain qualitative/quantitative insights into changes in the cellular proteome of two control (U-2 OS and N2) and two PPO-KO (R38/1 and W227/1) clones. MS-LFQ experiments were done in biological triplicates for each clone and the data from control and PPO-KO clones were combined for the final analysis (Fig. 3a and Table S3). Once again, MS-LFQ confirmed the complete absence of PPO in PPO-KO clones, while the expression levels of ALAS-1 were increased by approximately 77-fold compared to controls, thus validating western blotting data. Furthermore, increase in the ALAS-1 protein levels corroborates the inverse correlation with the cellular heme content as reported previously^6,49^. Apart from ALAS-1, expression levels of other enzymes of the heme biosynthesis pathway did not differ between PPO-KO and control cells. This also includes ferrochelatase that is believed to form a heterotetramer complex with PPO in mitochondria^38,39^. Our data thus suggest that the formation of the ferrochelatase/PPO complex is not critical for ferrochelatase stability.

In addition to ALAS-1 and PPO, our proteomic analysis of PPO-KO clones identified expression levels of 6 and 14 proteins to be significantly upregulated and downregulated, respectively (Table 2). Out of 14 downregulated proteins, only three use heme directly as the prosthetic group. Interestingly, eight of them are components of the mitochondrial electron transport chain (ETC) that is responsible for cell respiration and energy production. These ETC components include the cytochrome c electron carrier protein together with seven elements of oxidative phosphorylation (OXPHOS) complexes III and IV. Besides ALAS-1, additional 6 upregulated proteins are not known to be associated with heme or the heme metabolism so far and they play a role in glycolysis, protein and calcium transport, metal ion binding, regulation of GTPase activity, and DNA repair. Further studies are thus warranted to elucidate their connection(s) to the heme metabolism, including for example the little studied coiled-coil domain containing 32 protein with sixfold increased levels in PPO-KO cells.

**Table 2:** List of significantly dysregulated proteins in PPO-KO cells.

Protein name	Fold change	Gene name	Localization	Protein ID
**5-Aminolevulinate synthase 1**	**77.77**	**ALAS1**	**Mitochondrial matrix, cytosol, nucleus**	**P13196**
Coiled-coil domain containing 32	6.08	CCDC32	Nucleus	Q9BV29
Gametocyte-specific factor 1	3.39	GTSF1	Cytosol	Q8WW33
Voltage-dependent calcium channel subunit alpha-2/delta-1	2.22	CACNA2D1	Plasma membrane	P54289
Methylated-DNA–protein-cysteine methyltransferase	2.05	MGMT	Nucleus, membrane, cytosol, mitochondria	P16455
TBC1 domain family member 2A	2.01	TBC1D2	Cytosol, nucleoplasm, plasma membrane	Q9BYX2
Beta-enolase	1.85	ENO3	Cytosol, membrane	P13929
**Cytochrome b-c1 complex subunit 6, mitochondrial**	**0.63**	**UQCRH**	**Mitochondria, OXPHOS complex III**	**P07919**
**Cytochrome c oxidase subunit 6C**	**0.57**	**COX6C**	**Mitochondria, OXPHOS complex IV**	**P09669**
**Cytochrome c1, heme protein, mitochondrial**	**0.55**	**CYC1**	**Mitochondria, OXPHOS complex III**	**P08574**
**Cytochrome c oxidase subunit NDUFA4**	**0.52**	**NDUFA4**	**Mitochondria, OXPHOS complex IV**	**O00483**
Nurim	0.51	NRM	Nuclear membrane	Q8IXM6
**Cytochrome c oxidase subunit 6B1**	**0.50**	**COX6B1**	**Mitochondria, OXPHOS complex IV**	**P14854**
**Lanosterol 14-alpha demethylase**	**0.49**	**CYP51A1**	**Endoplasmic reticulum, membrane**	**Q16850**
**Cytochrome c oxidase subunit 3**	**0.46**	**MT-CO3**	**Mitochondria, OXPHOS complex IV**	**P00414**
DBH-like monooxygenase protein 1	0.45	MOXD1	Cytosol, membrane, secretory granule	MOXD1
**Cytochrome c oxidase subunit 6A1, mitochondrial**	**0.39**	**COX6A1**	**Mitochondria, OXPHOS complex IV**	**P12074**
ADP-ribosylation factor-like protein 6-interacting protein 6	0.38	ARL6IP6	Membrane	Q8N6S5
Cell division cycle protein 20 homolog	0.35	CDC20	Nucleus, cytosol	Q12834
**Cytochrome c**	**0.34**	**CYCS**	**Mitochondria, cytosol**	**P99999**
**Cytochrome B561 family member D2**	**0.26**	**CYB561D2**	**Endoplasmic reticulum, membrane**	**O14569**
**Protoporphyrinogen oxidase**	**NaN**	**PPOX**	**Mitochondrial matrix**	**P50336**

Value represents the fold change of the protein expression level relative to the control group. Heme-related proteins are shown in bold.

*NaN* not a number.

To corroborate mass spectrometric data, we used targeted western blotting to assess expression levels of ALAS-1, ferrochelatase, and PPO, which all are involved in heme biosynthesis, in 3 PPO-KO clones and 3 control cell lines (Fig. 3b and S5). In line with genotyping and enzymatic assays, antibody specifically recognizing PPO stained only control cell lines, revealing a positive band at the expected molecular weight of approximately 50 kDa, albeit at the detection limit of the assay. On the other hand, immunodetection of ALAS-1 indicated markedly increased expression in PPO-KO clones with virtually non-detectable signal in control cell lines. Finally, expression levels of ferrochelatase were below detection limit of our assays in all samples tested (data not shown).

Increased expression of ALAS-1 suggests the upregulation of the heme synthesis pathway in PPO-KO cells was caused by low levels of intracellular heme. To confirm this hypothesis, PPO-KO cells were treated with hemin for 20 h, followed by the analysis of ALAS-1 protein levels by Western blotting. While PPO-KO cells grown without hemin supplementation have detectable levels of ALAS-1, hemin treatment suppressed ALAS-1 expression levels below the detection limit of the method. These findings suggest that the heme synthesis pathway is downregulated by hemin imported by PPO-KO cells from culture media (Fig. S4c) and are in line with observed increase in intracellular heme concentrations determined by RP-HPLC (Fig. S4a).

### Mitochondrial respiratory complexes are disrupted in PPO-KO cells

**Figure 4:**
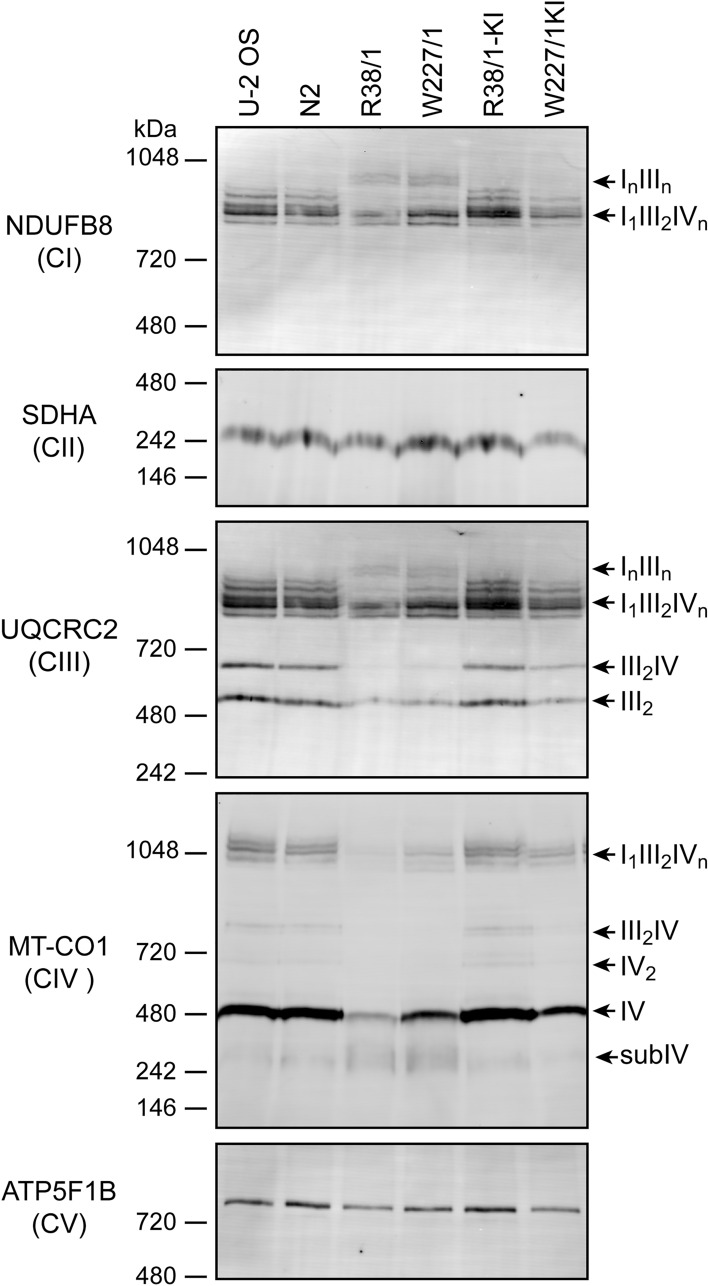
Analysis of OXPHOS (super)complexes. Protein complexes were extracted from cells by digitonin treatment and 20 µg of total proteins from each sample were separated by BN-PAGE and electroblotted onto a PVDF membrane. Antibodies specific for NDUFB8, SDHA, UQCRC2, MT-CO1 and ATP5F1B were used for detection of the complex CI, CII, CIII, CIV and CV, respectively. Signals of CII and CV were considered as loading controls.

Since protein levels of several OXPHOS components were downregulated in PPO-KO clones, we asked whether OXPHOS (super)complexes are affected. To this end, PPO-KO and control cells were lysed using digitonin and the protein (super)complexes of the ETC were analyzed by blue native polyacrylamide gel electrophoresis (BN-PAGE) followed by western blotting. Expression levels, assembly status and association into higher order (super)complexes for complexes I (CI), II (CII), III (CIII), IV (CIV) and V (CV) of OXPHOS were analyzed using subunit-specific antibodies against NDUFB8, SDHA, UQCRC2, MT-CO1, and ATP5F1B, respectively (Fig. 4 and S6).

In PPO-KO cells, we observed slightly increased levels of COX1 assembly intermediates (subIV), but lower levels of monomeric and dimeric forms of the complex IV and the fully assembled respirasome (I_1_III_2_IV_n_). Most striking difference was observed for the (super)complex III_2_IV, which was effectively absent in PPO-KO cells. The same pattern could also be observed using the antibody against CORE2 (UQCRC2), a subunit of the complex III. Less profound changes were observed for CI (using an antibody against the NDUFB8 subunit) with a drop in the levels of CI containing respirasome (I_1_III_2_IV_n_). Interestingly, the (super)complex I_n_III_n_ appeared only in PPO-KO cells, while it was not present in control cell lines (see also CORE2 signal for CIII). Accumulation of the I_n_III_n_ supercomplex may reflect the disturbed levels of I_1_III_2_IV_n_ and III_2_IV due to the lower availability of the complex IV, which makes some CIII portion available to form the I_n_III_n_ assembly. In contrast to the levels of complexes I, III and IV, the content of the complex II (using an antibody against SDHA) was not affected in PPO-KO cells. The same was true for the complex V (visualized with an antibody against the F_1_-β (ATP5F1B) subunit), which is in line with the fact that these complexes do not contain heme molecules and do not directly associate with the respirasome.

### Cell respiration and energy metabolism is compromised in PPO-KO cells

**Figure 5:**
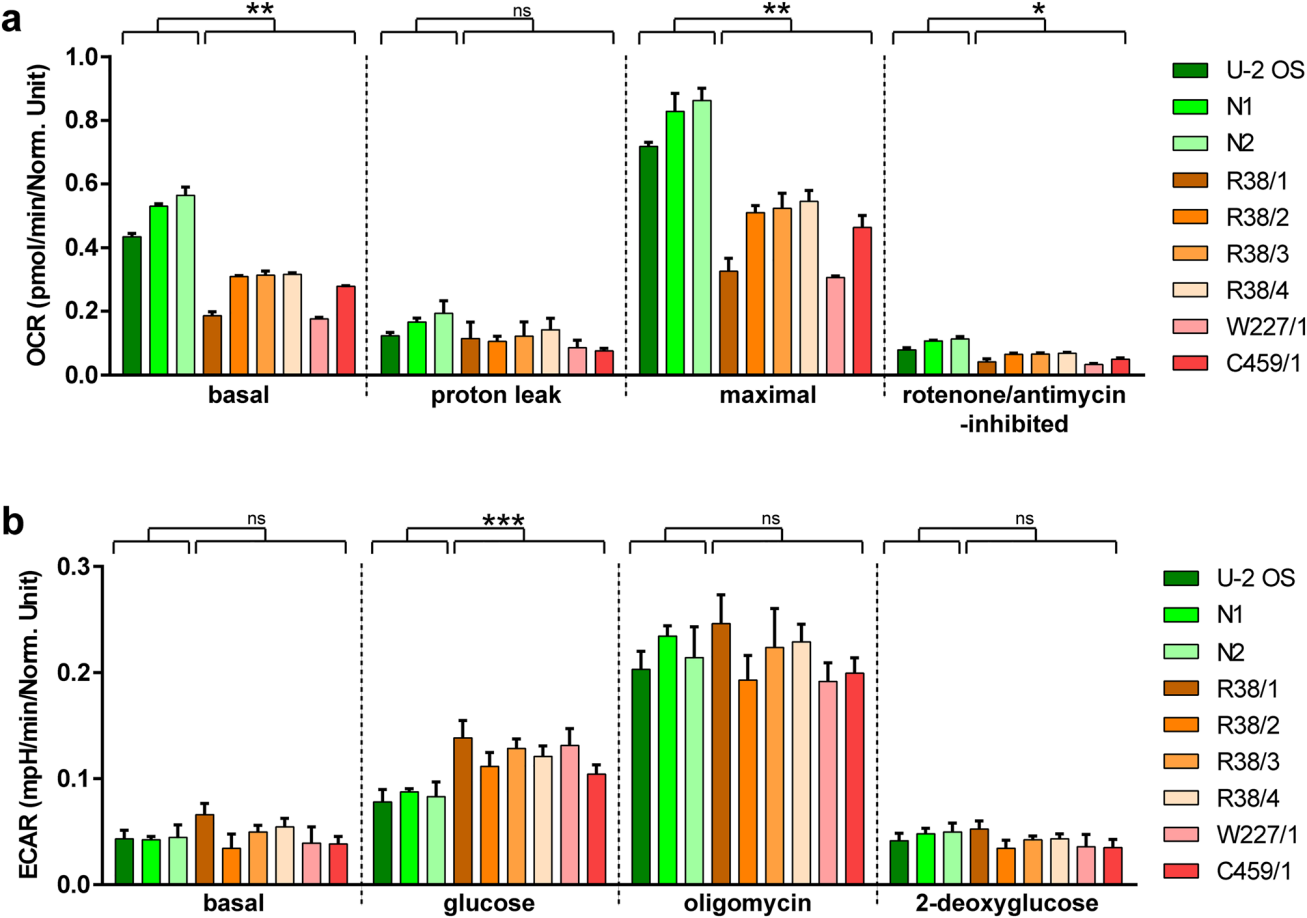
Analysis of mitochondrial respiration and glycolytic function. Oxygen consumption rate (**a**) and extracellular acidification rate (**b**) were estimated in PPO-KO clones and PPO knock-in cell lines by Seahorse extracellular flux analyzer. (**a**) ATP production, maximal respiration and spare respiratory capacity was driven by sequential treatment with 1 µM oligomycin, 1 µM CCCP, and 0.5 µM rotenone + 0.5 µM antimycin, respectively. (**b**) Basal glycolysis, glycolytic capacity and glycolytic reserve was induced by 10 mM glucose, 1 µM oligomycin, and 50 mM 2-deoxyglucose, respectively. Statistical significance was calculated by unpaired parametric t-test with Welch’s correction and assigned by ***(P < 0.001), **(P < 0.01), *(P < 0.05) and ns (non-significant; P > 0.05).

Given the disruption of the respiratory complexes, we further evaluated bioenergetic profile of PPO-ablated cells. Oxygen consumption rate (OCR) and extracellular acidification rate (ECAR) were measured in six PPO-KO clones and three control cell lines using the Seahorse XF96 Extracellular Flux Analyzer. OCR data pointed to a negative effect of PPO ablation on oxygen consumption. We detected notable decrease in basal as well as maximal respiration rate in all PPO-KO clones tested in comparison to control cell lines (Fig. 5a). Additionally, increased glycolysis, quantified trough ECAR, was found in PPO-KO clones when compared to control cell lines, although no major difference in glycolytic capacity and glycolytic reserve between PPO-KO and control cells was detected (Fig. 5b).

### Wild-type characteristics can be rescued upon transfection of PPO into PPO-KO cells

Rescue experiments were carried out to confirm that observed phenotypes in PPO-KO cell lines are linked to the absence of the PPO activity and not spurious off-target effects or results of clonal selection. To this end, R38/1 and W227/1 clones together with U-2 OS and N2 control cell lines were transfected with an expression plasmid harboring the PPO-GFP fusion with the wild-type PPO enzymatic activity. Using the GFP marker, PPO-GFP expressing cells were selected and knock-in (KI) cell lines established and further characterized.

**Figure 6:**
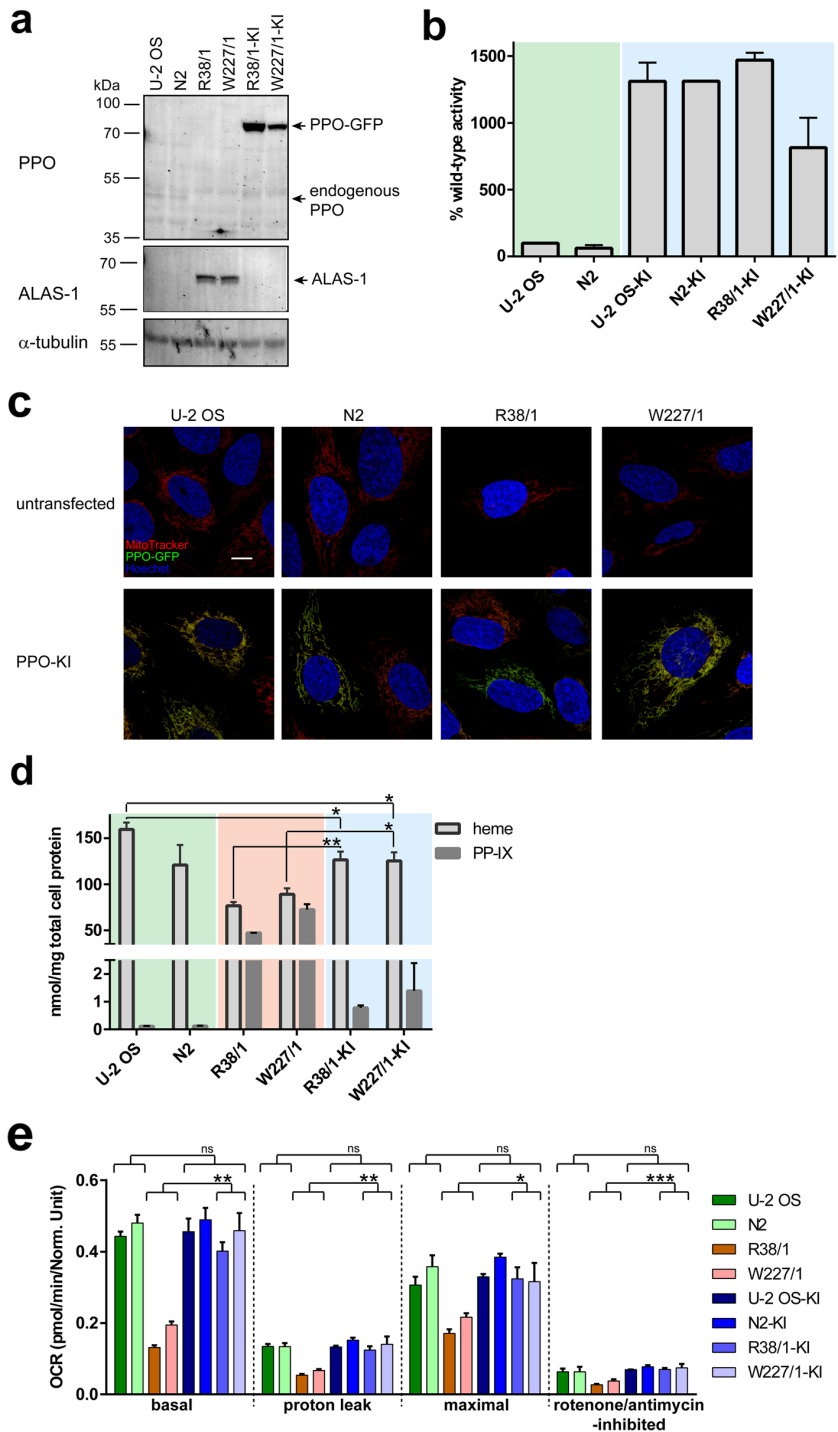
Characterization of PPO knock-in clones. R38/1, W227/1, U-2 OS, and N2 cell lines were transfected with the *PPOX*-GFP construct and corresponding knock-in cell lines denoted R38/1-KI, W227/1-KI, U-2 OS-KI and N2-KI, respectively. Control, PPO-KO and PPO-KI samples are highlighted by green, orange and blue background, respectively. (**a**) Expression levels of PPO-GFP and ALAS-1 were analyzed by Western blotting. Notice markedly higher PPO-GFP expression levels compared to endogenous PPO. Alpha-tubulin was used as a loading control. (**b**) PPO enzymatic activity in cell lysates was determined by following conversion of protoporphyrinogen IX to protoporphyrin IX. PPO oxidase activity is shown as ratio to the endogenous PPO activity in parent U-2 OS cells (100%). Data represent mean (± S.D.); n = 2. (**c**) Localization of PPO-GFP (green channel) was assessed by live-cell confocal microscopy. Mitochondria were visualized by vital staining using Mitotracker dye (red channel), cell nuclei were stained by Hoechst 33258 (blue channel). Scale bar 10 µm. (**d**) Intracellular concentrations of heme and protoporphyrinogen/protoporphyrin IX were determined by RP-HPLC. Data represent mean (± S.D.); n = 2. (**e**) The oxygen consumption rate was measured by Seahorse. ATP production, maximal respiration, and spare respiratory capacity were determined upon the addition of 1 µM oligomycin, 1 µM CCCP, and 0.5 µM rotenone + 0.5 µM antimycin A, respectively. Statistical significance was calculated by one-way ANOVA and assigned by ***(P < 0.001), **(P < 0.01), *(P < 0.05) and ns (non-significant; P > 0.05).

PPO-GFP expression levels and enzymatic activity in knock-in cell lines were evaluated by Western blotting and the PPO activity assay, respectively (Fig. 6a,b and S7). Both assays revealed pronounced expression of the PPO-GFP at levels well above the endogenous PPO expression. For example, the PPO enzymatic activity in knock-in clones was 10- to 15-fold higher compared to parent wild-type cell lines (Fig. 6b). Additionally, live-cell confocal microscopy confirmed mitochondrial localization of the overexpressed PPO-GFP, which colocalized with the mitochondria-specific Mitotracker Deep Red dye (Fig. 6c). Hence, the overexpression of the PPO-GFP fully restores functional PPO in knock-in cells.

Further characterization of knock-in clones validated that introduction of PPO-GFP largely recovered the parental cell phenotype. First, the heme levels in knock-in cell lines reverted close to the values of control cells (Fig. 6d). Interestingly, the PP-IX levels, while dropping approximately 50-fold to 1 nmol/mg cell proteins, still exceeded levels of PP-IX in control cells by ninefold (compare 0.116 ± 0.003 and 1.08 ± 0.44 nmol of PP-IX per mg cell proteins in parent and knock-in cells, respectively). We assume that increased levels of (likely) free protoporphyrin IX in knock-in clones result from the excessive PPO activity that can overload the iron-loading capacity of ferrochelatase in the subsequent heme biosynthetic step. Furthermore, overexpressed levels of ALAS-1 returned to original parental values (Fig. 6a), and, finally, the stability of mitochondrial (super)complexes (Fig. 4) as well as the oxygen consumption rate (Fig. 6e) were restored in knock-in clones.

## Discussion

Heme synthesis defects in humans are linked to a variety of metabolic diseases. Mutations in PPO, catalyzing the penultimate step of heme synthesis, can be manifested in the form of VP^23–25^, yet the underlying biochemical causes of VP development and consequences of PPO deficiency at the cellular level have not been fully resolved. Here we established and characterized a cellular model of PPO deficiency using the CRISPR/Cas9 system and our model was further extended by the reconstitution of the PPO enzymatic activity in knock-out cells.

PPO-KO cells do not reveal significantly decreased viability or proliferation; however, they accumulate high concentrations of protoporphyrinogen IX, and their intracellular levels of heme were permanently reduced approximately two-fold, suggesting that the cells likely offset the shortage by the uptake of extracellular heme. Despite this fact, we have found indices of heme deficiency and cellular adaptations to it. Firstly, PPO-KO cells upregulate ALAS-1, the first enzyme of heme biosynthesis that synthesizes aminolevulinic acid from succinyl-CoA and glycine, dramatically. This reaction represents the rate-limiting step with a negative feedback regulatory loop^6^. Indeed, high heme concentrations negatively influence transcription, translation, mRNA stability, protein stability and mitochondrial import of ubiquitously expressed ALAS-1^50–57^. Furthermore, one of the cellular consequences of decreased heme is compromised function of mitochondrial respiration. Since mitochondrial respiratory complexes, especially CIII and CIV contain heme, the physiological consequence of heme deficiency is destabilization of these complexes and the impaired oxygen consumption rate. This corresponds to the phenotype observed in PPO-KO cells that reveal compromised oxygen consumption, decreased CIII and CIV quantities and aberrant assembly of CI_n_CIII_n_ (super)complexes. It is interesting to note that the link between dysregulated heme levels accompanying VP and compromised function of OXPHOS complexes, mainly CIV, has been recently observed using a rabbit model of induced VP in vivo^58^, corroborating thus findings reported here. In their paper, Jerico et al. showed that the insufficient efficiency of derivatization of protoheme to heme *a* might be responsible for the decreased levels and the compromised function of CIV. As the deficiency of CIV in OXPHOS (super)complexes was also observed in our PPO-KO cells, further studies are warranted to address detailed molecular mechanisms at the cellular level.

In plants, PPO is a target of inhibitory herbicides that are among the most widely used herbicides in crop fields around the world^41,59,60^. Here, PPO inhibition leads to accumulation of protoporphyrinogen IX that acts as a potent photosensitizer. Plant illumination then triggers lipid peroxidation that compromise function of cellular membranes leading to death of plants^61,62^. Interestingly, PPO-targeting herbicides can also inhibit mammalian PPO and induce porphyria and cancerogenesis in mammals^63–65^.

Here we show that total absence of the PPO enzymatic activity is well tolerated under in vitro cell culture conditions. In contrast, it was found previously that PPO deficiency is incompatible with survival at the organismal level. In VP, the specific activity of PPO is typically decreased to approximately 50% of the control subjects. In fact, patients with the PPO activity below 20% of the healthy controls are extremely rare suggesting that very low/absent PPO activity may be lethal at the embryonic stage^66^. Indeed, disruption of the *PPOX* gene is embryonically lethal in experimental animals^40^ suggesting highly strict control of heme levels and enhanced uptake during development that would over time lead to depletion of heme in all compartments. Therefore, it is questionable whether long lasting enhanced demand of heme would significantly decrease survival of PPO-KO cells. Interestingly, the picture looks very similar for the components of mitochondrial OXPHOS machinery, where severe deficiency is not tolerated. Given our findings that PPO suppression disrupts OXPHOS, it is thus likely that respiratory insufficiency might be a significant factor in the etiology of PPO-linked VP. In line with this reasoning, compromised cellular respiration, namely decreased basal and maximal OCR, was observed in cells of porphyria patients with serious symptoms^67^.

In conclusion, we generated human cell lines deficient in the enzymatic activity of PPO and these cells can serve as a valuable tool to be used by the community to assess uptake, intracellular transport, and storage of heme as well as the regulation of its biosynthesis to answer outstanding questions surrounding heme (patho)physiology.

## Materials and methods

### Chemicals and antibodies

If not stated otherwise, reagents used in the study were purchased from Sigma-Aldrich (St. Louis, MO, USA). Antibodies (Ab) were used as follows: rabbit polyclonal anti-PPO Ab (0.5 µg/ml; CQA1363; Cohesion Biosciences, London, UK), mouse monoclonal anti-ALAS-1 Ab (0.1 µg/ml; sc-137093; Santa Cruz Biotechnology, Dallas, TX, USA), rabbit anti-α-tubulin Ab (0.02 µg/ml; ab18251; Abcam, Cambridge, UK), mouse monoclonal anti-NDUFB8 Ab (0.1 µg/ml; ab110242; Abcam, Cambridge, UK), rabbit polyclonal anti-UQCRC2 Ab (0.08 µg/ml; 14742-1-AP; Proteintech, Rosemont, IL, USA), mouse monoclonal anti-SDHA Ab (0.1 µg/ml; ab14715; Abcam, Cambridge, UK), mouse monoclonal anti-MT-CO1 Ab (1 µg/ml; ab14705; Abcam, Cambridge, UK), mouse monoclonal anti-ATP5F1B Ab (0.5 µg/ml; ab14730; Abcam, Cambridge, UK), donkey anti-rabbit secondary Ab conjugated to AlexaFluor-568 (0.3 µg/ml; A10042; Invitrogen, ThermoFisher Scientific, Waltham, MA, USA), goat anti-mouse secondary Ab conjugated to AlexaFluor-488 (0.3 µg/ml; A11029; Invitrogen), goat anti-mouse secondary Ab conjugated to AlexaFluor-680 (0.7 µg/ml; Invitrogen), goat anti-rabbit secondary Ab conjugated to IRDye800 (0.3 µg/ml; 611–132-002; Rockland Immunochemicals, Limerick, PA, USA).

### Cell lines

The U-2 OS human cell line, kindly provided by Pavel Hozák (IMG, Prague, Czech Republic), was grown in HEPES-supplemented high-glucose Dulbecco′s Modified Eagle′s medium (D-MEM; Sigma-Aldrich) supplemented with 10% fetal bovine serum (FBS) and 2 mM l-glutamine. Cells were cultivated under a humidified 5% CO_2_ atmosphere at 37 °C.

### CRISPR design

Specific guide RNAs (gRNAs) recognizing desired regions of the *PPOX* gene (Table S1) were designed using the online software CRISPOR Design Tool^43^ and synthesized commercially (Sigma-Aldrich). Complementary oligos of individual sgRNAs were annealed and sub-cloned into the BbsI-digested px458 plasmid (#48138, Addgene plasmid repository, Watertown, MA, USA) using the published protocol^68^. The identities of resulting px458-*PPOX*-CRISPR vectors were confirmed by Sanger sequencing.

### Heteroduplex analysis

Approximately 100,000–200,000 cells were harvested by trypsinization using 0.06% trypsin/0.02% EDTA. Cells were washed with phosphate buffered saline (PBS; 137 mM NaCl, 2.7 mM KCl, 1.4 mM KH2PO4, 8.1 mM Na2HPO4, pH 7.4), centrifuged at 300×*g* for 3 min and the cell pellet was re-suspended in 100 µl 60 mM NaOH. Cell suspension was heated at 95 °C for 30 min and the lysate was neutralized by the addition of 20 µl 1 M Tris–HCl, pH 6.8. The sequence of approximately 500 bps surrounding the CRISPR site in the *PPOX* gene was amplified by PCR using the cell lysate as a DNA template (0.75 µl per reaction). Amplification was carried out in the total volume of 10 µl with reaction mixture containing Phusion Flash High-Fidelity PCR Master Mix (ThermoFisher Scientific) and 500 nM primers listed in Table S2. PCR run parameters: initial denaturation (98 °C for 60 s), 30 cycles of denaturation (98 °C 3 s), annealing (65 °C for 5 s) and extension (72 °C for 30 s), finalized by extension step at 72 °C for 90 s. PCR products were separated by electrophoresis in 5% polyacrylamide gel using the TBE running buffer (89 mM Tris, 89 mM boric acid, 20 mM EDTA) and visualized with GelRed (Biotium, Fremont, CA, USA) under UV light.

### Transfection and establishing of CRISPR and knock-in clones

U-2 OS cells were seeded into a 24-well plate. The following day, cells were transfected at approximately 70% confluence by individual px458-*PPOX*-CRISPR plasmids using the GeneX*Plus* transfection reagent (ATCC, Manassa, VA, USA). Three days post-transfection, GFP-positive cells were selected using a BD FACSAria III Cell Sorter (BD Biosciences, San Jose, CA) and single-cell colonies were formed after low-density seeding (100 cells per 6-cm Petri dish precoated with 0.5% porcine gelatin) into the 1:1 mixture of conditioned and fresh growth media supplemented with 12.5 µg/ml hemin. Two months after cell sorting hemin supply was gradually reduced and cells were cultivated without hemin supplementation. To establish knock-in clones, PPO-KO clones as well as controls were transfected by a *PPOX*-GFP vector encoding human PPO C-terminally fused to GFP. Two days post-transfection, the medium was supplemented with 100 µg/ml Zeocin and cultures selected for three weeks. Finally, GFP-negative cells were removed from cultures using the BD FACSAria III Cell Sorter (BD Biosciences).

### PPO activity assay

The enzymatic activity of PPO in cell lysates was determined as an increase in a fluorescent signal upon conversion of non-fluorescent protoporphyrinogen IX to fluorescent protoporphyrin IX^69^. Approximately 4 × 10^6^ cells were harvested in 0.06% trypsin/0.02% EDTA, washed with PBS and lysed in 100 µl PBS/1.5% lauryl maltoside (Sigma-Aldrich) supplemented with the EDTA-free protease inhibitor cocktail (Roche, Basel, Switzerland). PPO activity in cell lysates was determined by incubating 10 µl of undiluted lysate with 10 µM porphyrinogen in the reaction buffer (100 mM KH_2_PO_4_, 0.3% (w/v) Tween-80, 5 mM DTT, 1 mM EDTA, pH 7.2) in the total volume of 20 µl in 384-well plates at 37 °C. The reactions were monitored continuously for 60 min with the fluorescence signal of the protoporphyrin IX product quantified using a CLARIOstar fluorimeter (BMG Labtech GmbH, Ortenberg, Germany) with excitation/emission wavelengths set at 410/632 nm, respectively. The data were normalized to the total protein content and analyzed using the GraphPad Prism software (GraphPad Software, San Diego, CA, USA).

### Genotyping

DNA template preparation and PCR amplification were carried out as described above. PCR products were inserted into the SmaI-linearized pUC19 vector using Blunt/TA Ligase (New England Biolabs, Ipswich, MA, USA). The ligation mixture was transformed into the DH5α bacterial strain, plasmid DNA isolated from individual colonies and inserts sequenced using primers designed for PCR amplification of a given *PPOX* region (Table S2).

### Fluorescence-based quantification of heme following iron chelation

The level of intracellular heme and porphyrins was determined as described previously^46^. Briefly, 2 × 10^6^ cells were thoroughly washed with PBS and lysed in 60 µl TBS/1% Triton-X100 supplemented with the EDTA-free protease inhibitor cocktail (Roche, Basel, Switzerland). Lysates were centrifuged at 2000×*g* at 4 °C for 5 min, and the protein content in supernatants was determined using the BCA protein assay (ThermoFisher Scientific). Supernatant samples were standardized to 6 mg of proteins/ml and 5 µl of samples were mixed with 200 µl of 2 M oxalic acid and warmed at 100 °C for 30 min. Cooled samples were pelleted by centrifugation at 2000×*g* for 15 min and porphyrin fluorescence was measured using the CLARIOstar fluorimeter (BMG Labtech GmbH, Ortenberg, Germany) with excitation and emission wavelength set to 405 nm and 600 nm, respectively. Data were analyzed in GraphPad Prism software (GraphPad Software, San Diego, CA, USA). Control samples mixed with water instead of oxalic acid were used for background subtraction.

### Determination of heme and PP-IX content using reversed-phase high-performance liquid chromatography (RP-HPLC)

Extraction of heme from cells was carried out according to the published protocol^70^ with modifications listed below. Cells were seeded at concentration 120,000 cells/ml on a 6-cm Petri dish. The following day, cells were extensively washed with 150 mM NaCl, scraped and centrifuged at 600×*g* at 4 °C for 4 min. The cell pellet was re-suspended in 200 µl 150 mM NaCl and further diluted with 800 µl acetonitrile. Samples were shaken at 700 rpm for 5 min with occasional vigorous vortexing and then centrifuged at 2 500xg at RT for 5 min in swing-out rotor. Pellets were re-suspended in 100 µl of a heme-extraction buffer (2 M HCl mixed with acetonitrile at the 1:4 ratio) and shaken vigorously at room temperature for 30 min. Samples were then centrifuged at 2 500×*g* at RT for 5 min, supernatants collected and analyzed by RP-HPLC as described below.

PP-IX extraction from cells was carried out according to protocol published previously^71^. Cells seeding and harvest was identical to procedure described above. The cell pellet was re-suspended in 20 µl H_2_O and cells were lysed by sequential addition of 2 µl 50% acetic acid and 60 µl dimethyl formamide supplemented with isopropanol at the 100:1 ratio. The cell lysate was centrifuged at 15,000×*g* for 5 min, the supernatant collected, the pellet was re-suspended in 50 µl dimethyl formamide/isopropanol mixture and centrifuged at 15,000×*g* for 5 min. The two supernatants were pooled, and the mixture was then analyzed by RP-HPLC.

Extracts were analyzed by RP-HPLC (Shimadzu, HPLC Prominence system) with a Kinetex^®^ 2.6 µm XB-C18 100 Å column (50 × 2.1 mm; Phenomenex, Torrance, CA, USA). The mobile phase A was 0.1% (v/v) trifluoroacetic acid (TFA) in water, the mobile phase B was 0.1% (v/v) TFA in acetonitrile. A linear gradient elution mode from 30 to 90% solvent B within 7 min with a flow rate of 0.6 ml/min was used. The injection volume was 20 µl. The UV/Vis absorbance detector was set to a wavelength of 400 nm for the analysis of heme. The excitation/emission wavelengths were set to 400/615 nm, respectively, for the analysis of PP-IX. The quantification of analytes was performed using calibration curves of known concentrations of human hemin (Recordati Rare Diseases, Puteaux, France) and protoporphyrin IX (Sigma-Aldrich) standards. The data were normalized to the protein content determined by the BCA protein assay (ThermoFisher Scientific). Injection dose of heme and PP-IX extract corresponded to 24 and 37 µg of total cell protein, respectively, representing thus the input of extraction procedure.

### Hemin treatment

Cells were seeded on a 6 cm-diameter Petri dish in the density 120,000 cells/ml in three sets and immediately treated with hemin dissolved in DMSO (final concentrations 2 and 8 µM). Control cells were treated by DMSO only. Following 20-h treatment, cells were harvested and intracellular heme and PP-IX concentrations quantified by RP-HPLC. In parallel, ALAS-1 protein levels were analyzed by Western blotting.

### Heme uptake

Uptake of heme was estimated by measurement of the uptake of Zn-PP analog according to the published protocol^47^. Briefly, cells were seeded at the density of 20,000 cells/well in a 96-well black gelatinized plate. After 24 h, 10 mM stock of Zn-PP dissolved in DMSO was diluted in growth medium to final concentration 60 µM Zn-PP, centrifuged at 7000×*g* for 20 min and supernatant was added to cells for 4 h. Then cultivation medium was thoroughly washed out by PBS and total fluorescence of Zn-PP was measured using the CLARIOstar fluorimeter with excitation/emission wavelengths set to 360 nm and 590 nm, respectively.

### Cell proliferation assay

The procedure carried out according to the published protocol^48^ with minor modifications. Cells were detached using 0.06% trypsin/0.02% EDTA, washed twice with PBS at 37 °C and diluted to 10^6^ cells/ml in PBS/0.004% EDTA containing 4 µM CFSE (Sigma-Aldrich). To reach high efficiency of labeling, EDTA was added to keep cells in monosuspension, and the CFSE stock solution (10 mM in DMSO) was diluted into PBS just < 1 min prior to incubation. Following 15-min incubation with continuous shaking at 37 °C, cells were pelleted at 300×*g* for 3 min, washed twice with cultivation media supplemented with 20% FBS, then diluted in standard cultivation media and seeded in the density 30,000 cells/ml (four sets for each cell line analyzed). Three hours after seeding the medium was exchanged to remove remaining extracellular CFSE. At defined time points, individual cell sets were harvested by trypsin/EDTA and the concentration of intracellular CFSE was determined with a LSRFortessa flow cytometer (BD Biosciences) using PBS/0.5% gelatin supplemented by 1 µg/ml Hoechst 33258 to identify live cells. Approximately 30,000 cells were analyzed by the FlowJo software (FlowJo, LLC, Ashland, OR).

### Proteomic analysis

Cells were harvested by scraping after thorough PBS wash. Using colorimetric Bradford assay aliquots of cell pellets containing 100 μg protein were frozen in liquid nitrogen and kept at −80 °C until further analysis. MS LFQ was performed on in-solution digested lysates processed in biological triplicates. Briefly, cell pellets were solubilized by sodium deoxycholate (final concentration 1% (w/v)), reduced with TCEP [tris(2-carboxyethyl)phosphine], alkylated with MMTS (*S*-methyl methanethiosulfonate), digested sequentially with Lys-C and trypsin and extracted with ethylacetate saturated with water as described^72^. Samples were desalted on Empore C18 columns, dried in Speedvac and dissolved in 0.1% trifluoroacetic acid + 2% acetonitrile. About 0.5 µg of peptide digests were separated on 50 cm C18 column using 2.5 h elution gradient and analyzed in a DDA mode on Orbitrap Exploris 480 (ThermoFisher Scientific) mass spectrometer. Resulting raw files were processed in MaxQuant (v. 1.6.17.0) with label-free quantification (LFQ) algorithm MaxLFQ. The search was performed at 0.01 FDR levels using human fasta file from UniProt (release 2021_01). Downstream analysis and visualization were performed in Perseus software^73^.

### SDS-polyacrylamide electrophoresis (SDS-PAGE) and immunodetection

1.5 × 10^6^ cells were harvested by 0.06% trypsin/0.02% EDTA, washed with PBS and lysed in 75 µl of TBS/0.5% Triton-X100 supplemented with the EDTA-free protease inhibitor cocktail. After centrifugation at 2000×*g* at 4 °C for 5 min, the supernatant was mixed with the Laemmli buffer supplemented with 100 mM dithiothreitol and heated to 95 °C. Protein content in samples was determined by the BCA protein assay (ThermoFisher Scientific) and equal amounts of protein samples were separated by SDS-PAGE and then electroblotted onto a PVDF membrane in Tris-CAPS/10% methanol buffer (Bio-Rad Laboratories) using a Trans-Blot SD Semi-Dry Transfer Cell (Bio-Rad Laboratories). The membrane was incubated in a blocking buffer (5% bovine serum albumin/PBS/0.05% Tween-20 for anti-PPO Ab; 5% non-fat dry milk/PBS/0.05% Tween-20 for other Ab) for 45 min and then stringently washed with PBS/0.05% Tween-20. Primary antibodies diluted in 5% bovine serum albumin/PBS/0.05% Tween-20 were applied on a membrane at 4 °C overnight and following stringent washing steps, further incubated with secondary antibodies diluted in the blocking buffer at room temperature for 2 h. The fluorescence signal was scanned by a Typhoon FLA9500 imager (GE Healthcare Life Sciences, Chalfont St Giles, UK) equipped with 473 nm and 532 nm lasers, and LPB Y510 and LPG 0575 filters. Signal quantification was done in Quantity One software (Bio-Rad Laboratories) using linear regression. Images were processed in Adobe CS4 Photoshop software (Adobe Systems, San Jose, CA).

### Blue native polyacrylamide gel electrophoresis (BN-PAGE)

Cells were washed by PBS, scraped and centrifuged at 600×*g* for 5 min. Cell pellets equal to 500 µg of total protein were suspended in 70 µl lysis buffer (50 mM NaCl, 50 mM imidazole, 2 mM ɛ-aminocaproic acid, 1 mM EDTA, pH 7.0). Protein complexes were solubilized by digitonin (Sigma-Aldrich) at the final concentration of 6 g digitonin/g total protein. Samples were incubated at 4 °C for 20 min and cell debris removed by centrifugation at 30,000×*g* for 30 min at 4 °C. Samples were supplemented by glycerol (10%), ɛ-aminocaproic acid (50 mM) and Coomassie Brilliant Blue G-250 (0.5%; Serva, Heidelberg, Germany). Protein complexes (20 µg of total cell proteins) were separated by BN-PAGE in 4–13% polyacrylamide gradient gels as described earlier^74^ using a Mini-PROTEAN III apparatus (Bio-Rad Laboratories, Hercules, CA, USA). Immunodetection procedure and the list of used antibodies was virtually identical to that listed above and published previously^75,76^. The modification included the use of different antibodies: anti-NDUFB8 Ab and anti-UQCRC2 Ab were used for the detection of complex I and III, respectively.

### Measurements of respiration and glycolysis

The Seahorse XF96 Extracellular Flux Analyzer (Agilent) was used to assess OCR and ECAR. Cells were seeded in poly-l-lysin-coated XF96 cell culture microplates (Agilent) at the density of 15,000 cells per well and cultivated overnight in the standard growth medium. Next day, the growth medium was replaced by the assay medium (DMEM with 2 mM glutamine but without glucose, carbonate and a pH indicator dye, pH 7.4 at 37 °C). For OCR measurements, the assay medium was additionally supplemented with pyruvate (1 mM), glucose (10 mM) and fatty acids-free bovine serum albumin (0.2% w/v). The plates were incubated in a 37 °C incubator without CO_2_ for 1 h, followed by the measurement in the Seahorse instrument. For OCR, the wells were consecutively injected with oligomycin (1 µM f.c.), carbonyl cyanide 3-chlorophenylhydrazone (CCCP; 1 µM f.c.) and rotenone/antimycin A (both 0.5 µM f.c.). For ECAR measurements, glucose (10 mM f.c.) was injected first, followed by oligomycin (1 µM f.c.; Cayman Chemicals, Ann Arbor, MI, USA) and then 2-deoxyglucose (50 mM f.c.; Cayman Chemicals). Once measurements were completed, the cell nuclei were stained by Hoechst 33342 and the number of cells per well was determined using an imaging plate reader (Molecular Devices, San Jose, CA, USA). Measured extracellular flux rates were normalized to the cell number.

### Intracellular localization of PPO

Intracellular localization of overexpressed PPO-GFP construct was determined by confocal microscopy. Briefly, a day before imaging, cells were passaged onto glass cover slips. Immediately prior to imaging, cells were successively treated at 37 °C with 50 nM Mitotracker Deep Red FM (ThermoFisher Scientific) for 25 min and then with Hoechst 33258 (100 µg/ml) for 30 min. Coverslips were immersed in a FluoroBrite DMEM medium (ThermoFisher Scientific) and cells were placed into a humidity chamber (OKOlab, Puzzuoli, Italy) and imaged by a confocal microscope (DMI8, Leica Microsystems, Wetzlar, Germany) using a 63× water immersion objective. Scans were analyzed by the ImageJ 1.53k software^77^ (National Institutes of Health, Bethesda, MD, USA) and Adobe Photoshop CS4 software (Adobe Systems, San Jose, CA, USA; https://www.adobe.com/products/photoshop.html).

## Supplementary Information

Supplementary Information.

Supplementary Table S3.

## Data Availability

All data generated or analysed during this study are included in this published article (and its Supplementary Information files).

## Abbreviations

ALAS: 5-Aminolevulinic acid synthase
BN-PAGE: Blue native polyacrylamide gel electrophoresis
CFSE: Carboxyfluorescein succinimidyl ester
CRISPR: Clustered regularly interspaced short palindromic repeats
ECAR: Extracellular acidification rate
ETC: Electron transport chain
FBS: Fetal bovine serum
HCP-1: Heme carrier protein 1
KI: Knock-in
KO: Knock-out
MS-LFQ: Mass spectrometry-label free quantification analysis
OCR: Oxygen consumption rate
OXPHOS: Oxidative phosphorylation
PPO and *PPOX*: Protoporphyrinogen oxidase IX
PP-IX: Protoporphyrinogen/protoporphyrin IX
RP-HPLC: Reversed-phase high-performance liquid chromatography
SDS-PAGE: SDS-polyacrylamide electrophoresis
VP: Variegate porphyria
Zn-PP: Zn-protoporphyrinogen IX
