# Supplementary material for: Generation and characterization of human U-2 OS cell lines with the CRISPR/Cas9-edited protoporphyrinogen oxidase IX gene
Source: Sci Rep. 2022 Oct 12;12:17081. doi: 10.1038/s41598-022-21147-x (PMC9556554; doi:10.1038/s41598-022-21147-x)

Supporting information

Generation and characterization of human U-2 OS cell lines with the CRISPR/Cas9-edited protoporphyrinogen oxidase IX gene

Zora Novakova[1]*, Mirko Milosevic[2,3], Zsofia Kutil[1], Marketa Ondrakova[1], Barbora Havlinova[1], Petr Kasparek[4], Cristian Sandoval-Acuña[5], Zuzana Korandova[6,7], Jaroslav Truksa[5], Marek Vrbacky[6], Jakub Rohlena[2], Cyril Barinka[1]*

Table S1

Table S2

Table S3 (an Excel file)

Figure S1

Figure S2

Figure S3

Figure S4

Figure S5

Figure S6

Figure S7

Table S1

Nucleotide sequences of designed guide RNAs specific for the *PPOX* gene.

CRISPR/Cas9 target site	Designed guide RNA sequence
Arginin 38	5´-GTGGAGAGCAGTGAGCGTCT-3´
Tryptophan 227	5´-GACCTCCACGAAGTGACCAC-3´
Cystein 459	5´-GTTAATGACTGTATAGAGAG-3´

Table S2

List of primers used for heteroduplex identification and genotyping of *PPOX* alleles.

CRISPR/Cas9 target site	Primer name-orientation	Sequence
Arginin 38	R38-forward	5’-AAGGTGAGTGCTCCACTTGTGCCAG-3’
	R38-reverse	5’-GCTCAGAAACCTGCTCTCCACATGC-3’
Tryptophan 227	W227-forward	5’-TTGTAGAGATGCAGTTTCACTATGTTGGCC-3’
	W227-reverse	5‘-CTAGGATTCTGGGGTAGCCCATGTC-3’
Cystein 459	C459-forward	5’-GGAAAACAGCTGGGCTGAGGAGG-3’
	C459-reverse	5‘-CTGGGCATTTTCTGCCTATGCTGGAATA-3’

Table S3 (an Excel file)

MS-LFQ analysis of protein expression levels in PPO-KO cells. Data of all proteins identified are shown in the Table S3 (separate file). The difference between the control groups (average of U-2 OS and N2) and the PPO-KO groups (average of R38/1 and W227/1) is expressed as log2(PPO-KO)-log2(control). An imputation method was employed for proteins with the MS signal below the detection limit of the method in one of analyzed samples.

Fig. S1. Raw images of agarose gels from analysis of *PPOX* allele heteroduplexes from CRISPR/Cas9 edited cell lines.

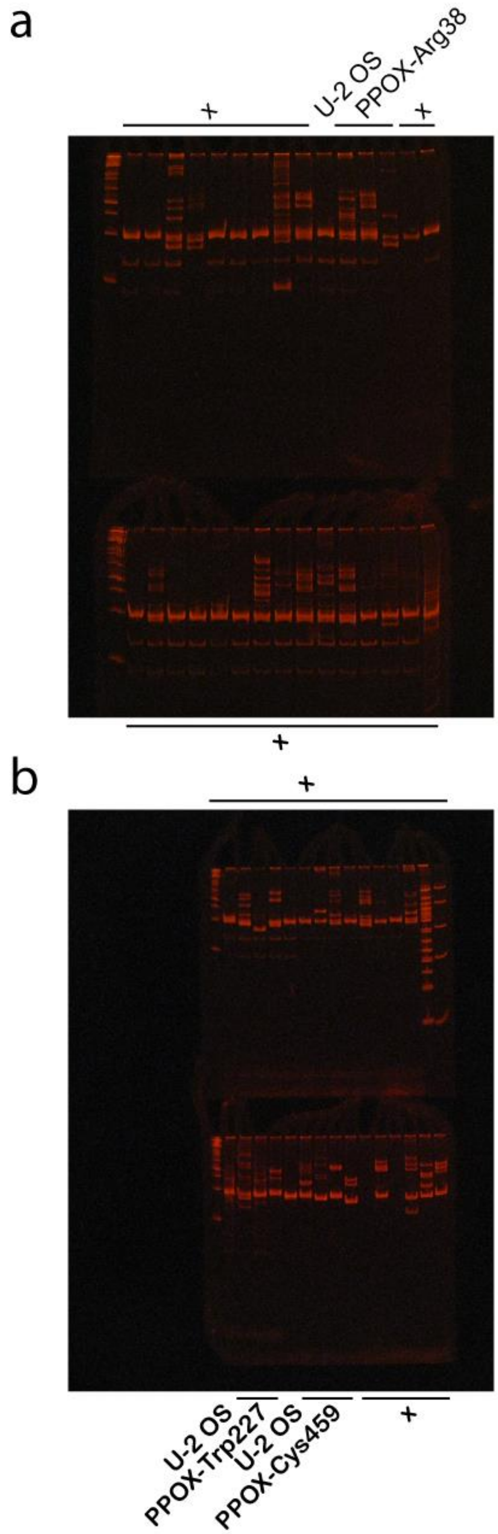

Fig. S2. Analysis of the heme content in cell extracts. The example of an HPLC chromatogram of the U-2 OS cell lysate. Heme was detected by the absorbance at 400 nm in 4.7 min.

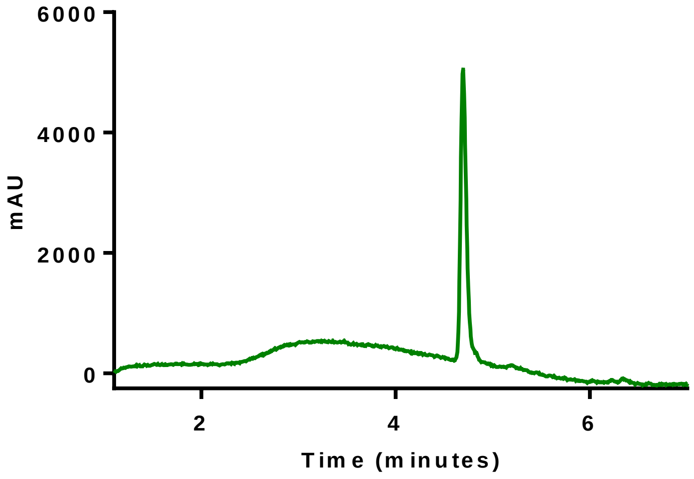

Fig. S3. Analysis of PP-IX content in cell extracts. The example of HPLC chromatogram of the W227/1 cell lysate. PP-IX was detected by fluorescence (λex/λem = 400/ 615 nm) in 6.5 min.

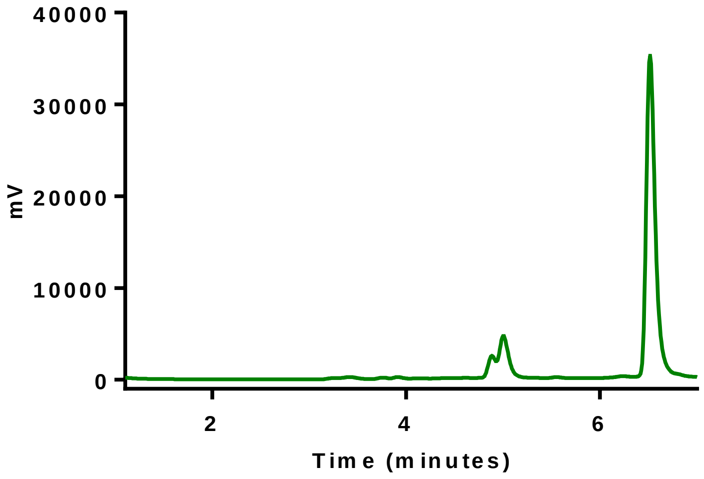

Fig. S4. Characterization of hemin-treated wild-type and PPO-KO cells. The level of intracellular heme (a) and protoporphyrinogen/protoporphyrin IX (b) was determined by RP-HPLC in cells treated with hemin for 20 hours. (c) Expression levels of ALAS-1 were determined by Western blotting of cell lysates separated by SDS-PAGE. Alpha-tubulin was used as a loading control. Raw unprocessed images showed on the right side.

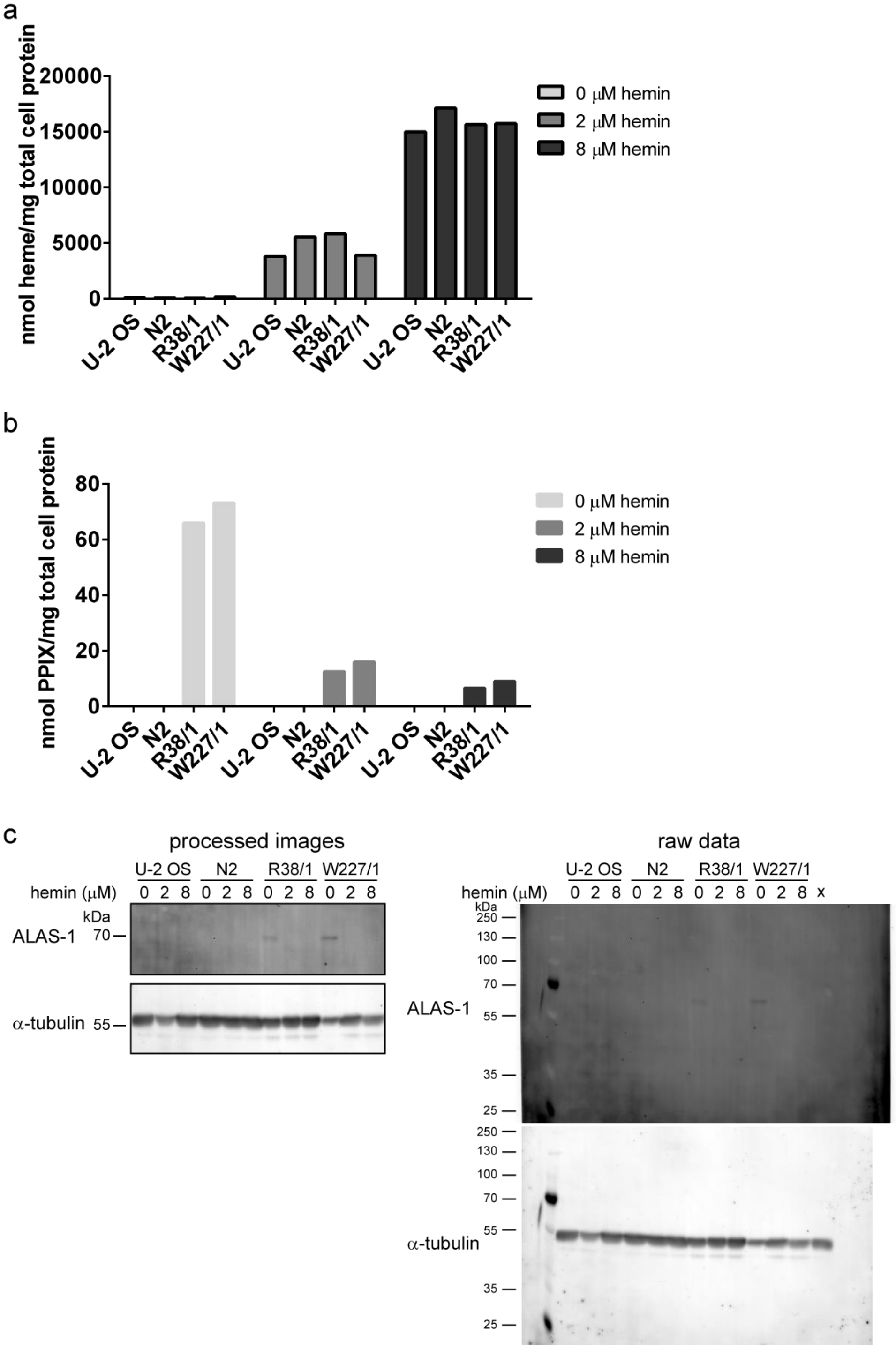

Fig. S5. Raw unprocessed from analysis of protein levels in PPO-KO cell lines.

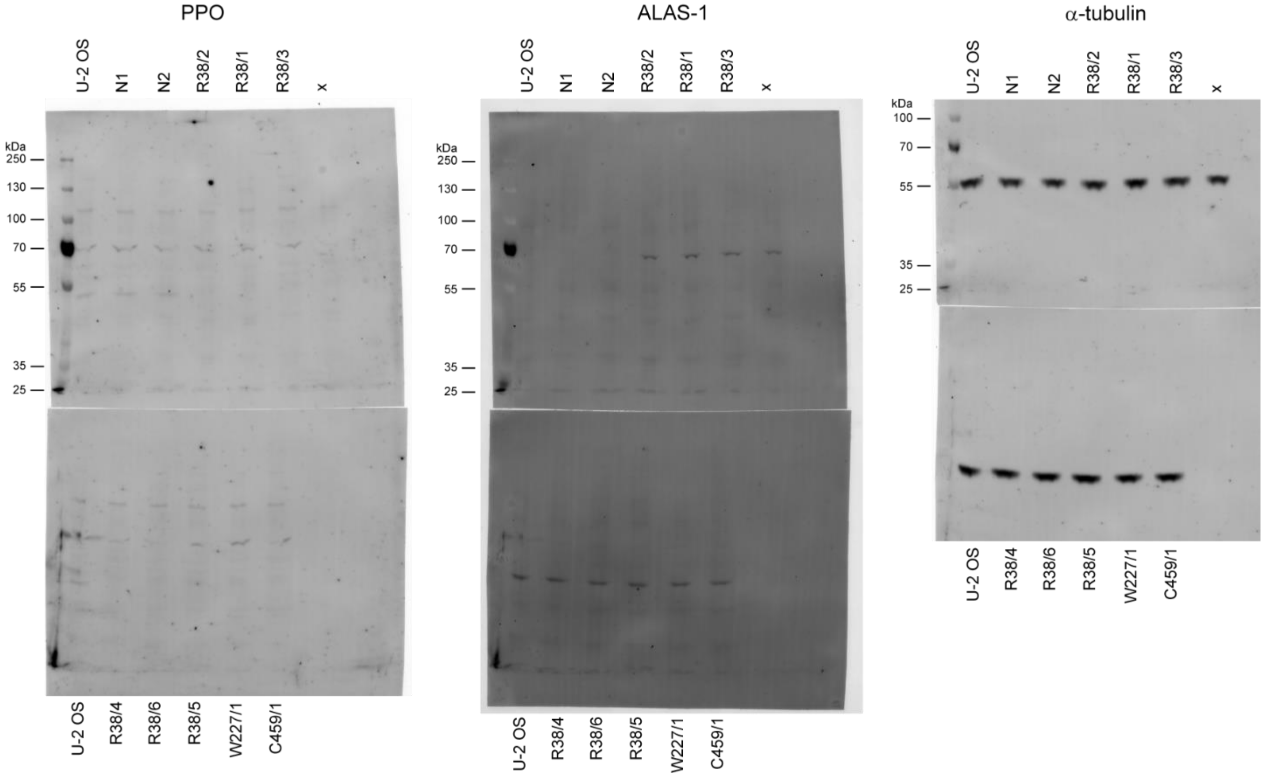

Fig. S6. Uncropped and unprocessed western blot images shown in Fig. 4 of the main text. Individual OXPHOS complexes were separated by BN-PAGE and visualized with antibodies specific for NDUFB8, SDHA, UQCRC2, MT-CO1 and ATP5F1B that were used for detection of the complex CI, CII, CIII, CIV and CV, respectively.

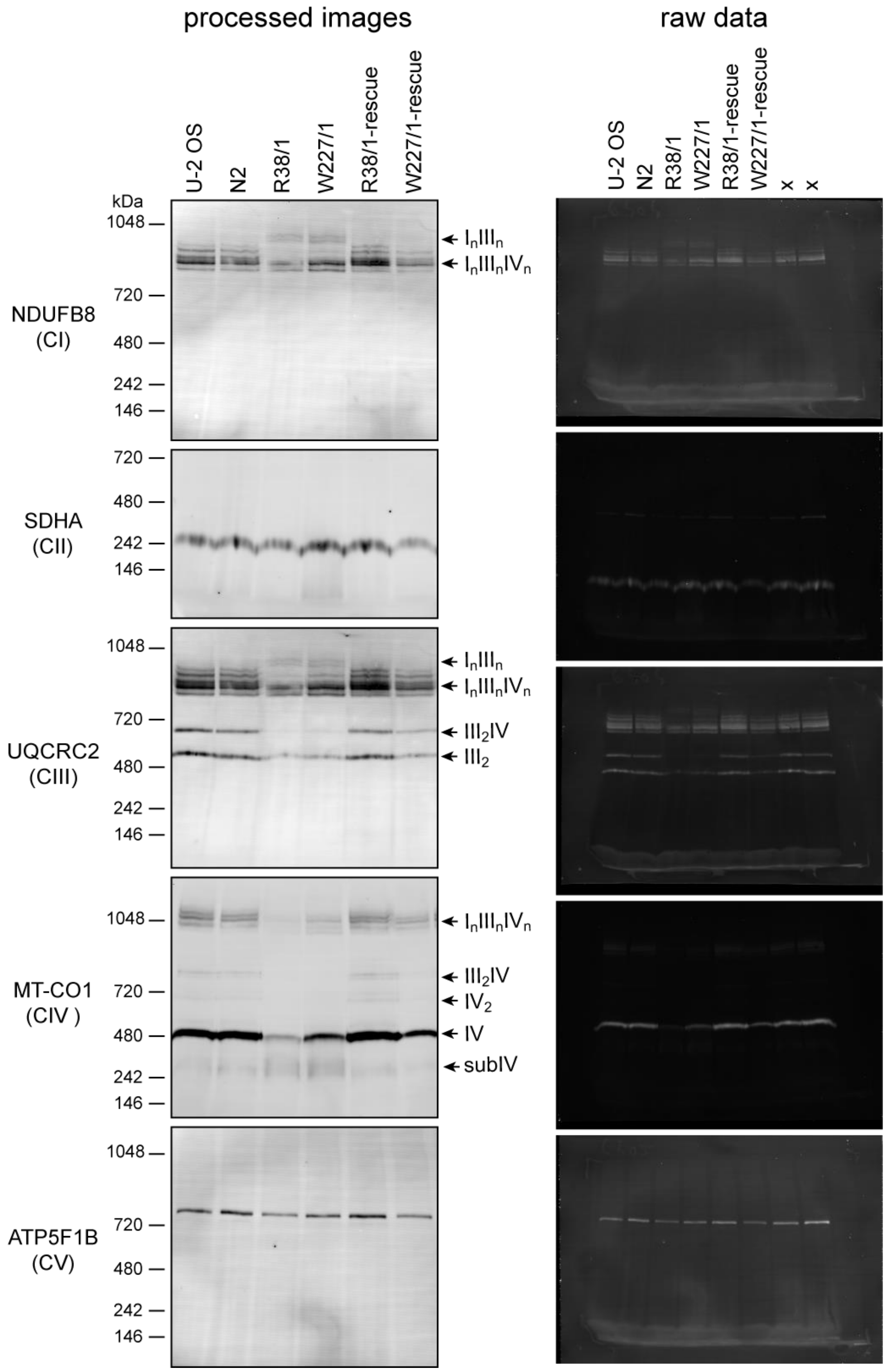

Fig. S7. Raw images of the analysis of expression levels in PPO knock-in clones.

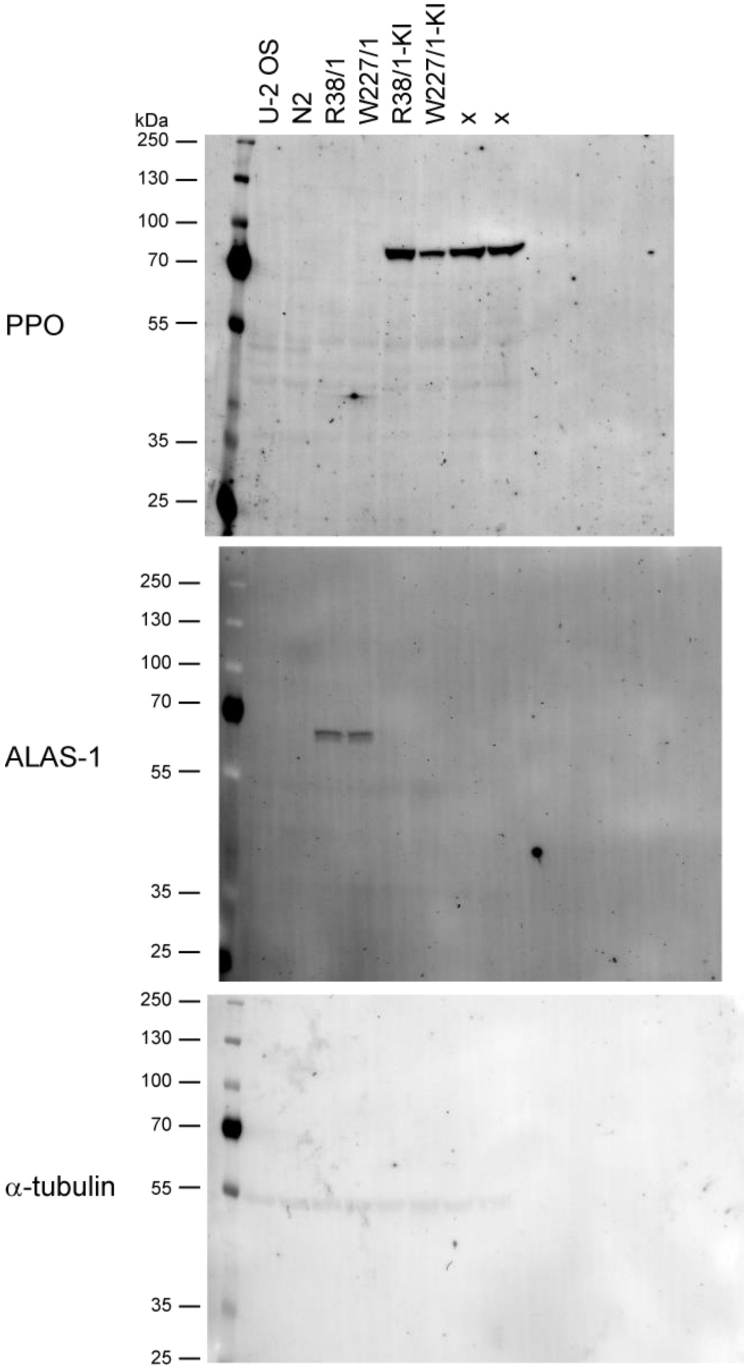

Supplement: Supplementary file 1 — Supplementary Information. [file 41598_2022_21147_MOESM1_ESM.pdf]
